# Sensitivity and specificity of standardised allergen extracts in skin prick test for diagnoses of IgE-mediated respiratory allergies

**DOI:** 10.1186/s13601-019-0248-9

**Published:** 2019-02-18

**Authors:** Nicola Wagner, Michael Rudert

**Affiliations:** 1 Department of Dermatology, University Hospital of Erlangen, Friedrich-Alexander-University Erlangen-Nürnberg (FAU), Ulmenweg 18, 91054 Erlangen, Germany; 2 Allergopharma GmbH & Co. KG, Hermann-Körner-Str. 52, 21465 Reinbek, Germany

**Keywords:** IgE-mediated allergy, Allergen extract, Skin prick test, Specific IgE, ROC analysis, Inhalant allergy

## Abstract

**Background:**

Skin prick tests (SPTs) are essential for the diagnosis of IgE-mediated allergy and are influenced by extract quality, biological potency and concentration of allergen.

**Methods:**

In this open multicentre study 431 patients, aged 18–64 years were enrolled. Patients had a history of IgE-mediated allergy and a sensitisation (previous positive SPT of any manufacturer) against at least one of the investigated allergens: 6-grass pollen, house dust mite, birch and mugwort pollen. In our study, these allergens were tested in five concentrations each. To establish the optimal trade-off between sensitivity and specificity, the area under the receiver operating characteristic (ROC) curve was estimated by comparing the outcome of the SPT with three methods referred to as ‘reference methods’ (specific IgE, clinical case history and a previous SPT).

**Results:**

For all allergens and reference methods, the area under the ROC curves were highly significant (p < 0.001). Specific IgE reference method resulted in the largest area under the curve (AUC) for all allergens (0.80–0.90) followed by previous SPT (0.70–0.87) and case history (0.65–0.74). Sensitivity of SPT increased with increasing concentration and specificity decreased. For all allergens, compared to specific IgE, the highest sensitivity (specificity at least 80%) was observed for the SPT solution of 50,000 Standardised Units (SU)/mL (grass pollen, birch pollen, house dust mite and mugwort).

**Conclusion:**

In this study, with a large number of patients, it was demonstrated that clinical case history, previous SPT and specific IgE measurement could all be used as reference methods for the assessment of sensitivity/specificity of SPT solutions. The comparison of SPT with specific IgE resulted in the largest AUC. The highest sensitivity was observed for the SPT solution of 50,000 SU/mL.

*Trial registration* EudraCT: 2006-005304-14.

## Background

The specific diagnosis of IgE-mediated allergy to aeroallergens is usually based on the correlation between clinical symptoms and medical history supplemented by diagnostic tests [1]. The clinical history is the basis for suspecting a type I IgE-mediated allergy, while the diagnostic tests are used to confirm or exclude the presence of specific IgE antibodies. Skin prick tests (SPTs) are useful as a single modality for demonstrating an IgE-mediated mechanism causing clinical symptoms [2]. To judge whether a positive SPT is of clinical relevance, it is important to understand the different factors that can influence the results of skin prick testing. To interpret the outcome of SPT correctly, and understand the test results, knowledge about sensitivity and specificity of the individual extracts is important. Quality of composition and content of allergens, especially of major allergens, in prick test solutions are mandatory in order to obtain reliable results.

A wide variety of factors may influence the result of SPTs. These include the particular SPT technique used; the site used for skin prick testing, the time of day, the age, sex, and race, and concomitant drug treatment [2]. The quality of allergen extract is of main significance as a wide variation in composition and allergen content between allergen extracts from different manufacturers exists. Biological examinations of biological units or content in micrograms of major allergens should be applied [1, 3].

Allergen extracts for SPT are native allergens obtained by extraction from the relevant biological material such as pollen, mites, animal epithelia and moulds. To achieve batch-to-batch consistency, in vitro standardisation of allergen extracts and determination of the biological activity are of crucial importance for the reliability of the test system.

Standardisation and composition alone do not necessarily ensure that allergen extracts used for skin prick testing are of an appropriate concentration to minimise the possibility of false positive and false negative skin reactions [1, 4, 5]. The diagnostic value of an allergen extract can only be assessed with respect to a population consisting of sensitised (true positive) and non-sensitised (true negative) patients. The Guideline on Clinical Evaluation of Diagnostic Agents recommends comparing the results yielded by the investigational diagnostic agent with the results of the so-called ‘standard of truth’ [6]. For allergen skin prick test solutions no such ‘standard of truth’ is defined. In current medical practice, analyses for circulating specific IgE antibodies in serum as well as the clinical history and SPT are considered to be standard methods to differentiate sensitised from non-sensitised patients [7, 8], and to confirm the clinical relevance of the allergen in question. In this study, each of these three reference methods was chosen as reference for the assessment of sensitivity and specificity of the SPT solutions. The objective of this multicentre study was to identify the most appropriate concentration for standardised prick extracts with regard to sensitivity and specificity in allergic patients. The following allergens for SPT were investigated: grass pollen, house dust mite, birch pollen, and mugwort pollen. These allergen extracts are currently available in several European countries.

## Methods

### Patients

Patients aged 18–64 years were enrolled in the study. All patients had a history of IgE-mediated allergy against at least one of the four investigational allergens. Further, the patients had a sensitisation to at least one of the investigational allergens evaluated by a SPT performed within 12 months before enrolment in this study (referred to as ‘previous SPT’), irrespective of the manufacturer used.

### Skin test material

Four different SPT solutions were tested: 6-grass pollen mixture (*Holcus lanatus*, *Dactylis glomerata*, *Lolium perenne*, *Phleum pratense*, *Poa pratensis*, *Festuca elatior*), house dust mite (*Dermatophagoides pteronyssinus*), birch pollen (*Betula verrucosa*) and mugwort pollen (*Artemisia vulgaris*). The test products were provided in vials containing five different concentrations of each allergen increasing in threefold steps of 5555 Standardised Units (SU)/mL, 16,666 SU/mL, 50,000 SU/mL, 150,000 SU/mL and 450,000 SU/mL. Histamine dihydrochloride (10 mg/mL) was used as positive control and saline solution as negative control solution. All SPT solutions and positive and negative controls were manufactured by Allergopharma GmbH & Co. KG, Reinbek, Germany.

### Study design

The study was conducted at six centres in Germany as a multicentre phase III/IV study during January to July 2008. SPT solutions were applied in a blinded way according to allergen, concentration, and negative and positive control. Neither the patient nor the investigator knew which solution was tested at which area on the volar sides of the forearms. There were two visits. Patients were invited for a first assessment to evaluate their eligibility including allergy history and documentation of a previously positive SPT reaction (a SPT of any manufacturer performed within the last 12 months prior to study entry). If patients were found eligible, the skin prick testing with all four investigational allergens in five different concentrations was performed at the following visit.

To avoid a drug induced influence on the SPT results, antihistamines, corticosteroids, mast cell stabilisers and drugs with concomitant antihistaminic effect were not used one to six weeks before prick testing—depending on the medication used [1].

Written informed consent was obtained from the patients before they were enrolled in the study, and the study was performed in accordance with good clinical practice. The principal ethics committee of Hessen (Trial registration: EudraCT: 2006-005304-14), local ethics committees of the participating centres and the regulatory authority in Germany (Paul-Ehrlich-Institute) approved the study.

### Skin prick test

SPTs were performed on the volar sides of both forearms. Each investigational allergen was tested in five different concentrations. The investigational allergens as well as the different solutions of positive and negative controls were applied blinded. The test areas were numbered by means of a suitable skin marker. The test areas had a minimum distance of 3 cm to each other [9]. The skin was pricked lightly and quickly vertically through the drop of the SPT solution by means of a microlancette (Allergopharma Prick Test Lancets). For each prick a new microlancette was used.

The test solution was removed immediately after the SPT by laying an absorbent paper towel on the skin prick area and carefully pressing it on the skin, without blending the different dilutions. The wheals outlines were read after 15–20 min. The wheal outline was taken off from the patient’s skin and documented by sticking them into the patient’s record sheet using a broad piece of translucent tape, which allows to preserve the original wheal area [10]. For assessment of a positive SPT reaction, the wheal had to be ≥ 3 mm in diameter. A valid SPT result also required positive histamine reaction (≥ 3 mm) and a negative saline control reaction (< 3 mm) [11]. The evaluation of the respective wheal area was carried out by using a validated digital image analysis system (based on software solution “analySIS”, OLYMPUS Soft Imaging Solutions GmbH; Münster, Germany). The patients had to stay in the physician’s practice for at least 30 min after measuring the prick test result.

### Estimation of sensitivity and specificity

The optimal diagnostic concentrations of the investigational SPT solutions, defined as optimal trade-off between sensitivity and specificity, was identified by comparing the outcome of the SPT with circulating specific IgE (ImmunoCAP™, Phadia, Uppsala, Sweden), the case history and a previous SPT (performed within 12 months before the study). These three methods were used as comparators because no’standard of truth’ according to ‘Points to Consider on the Evaluation of Diagnostic Agents’ (CPMP/EWP/1119/98) [6] has been defined for the validation of SPT solutions.

### Statistics

Receiver Operating Characteristic (ROC) analyses were carried out to investigate the appropriateness of the three used reference tests by detecting an optimal diagnostic concentration. Using this method, it was investigated whether a positive SPT result is a valid test for the diagnosis of IgE-mediated allergy. It was done for each allergen and each reference method tested. The null hypothesis, that the estimated AUC (Area Under Curve) equals 0.5 was tested confirmatively (Bonferroni procedure: α = 0.05/12 under consideration of the multiplicity problem resulting from usage of the data from the same patient for the determination of the ROC curves for four allergens and three different reference methods).

The optimal diagnostic concentration for each allergen was investigated by determination of sensitivity and specificity for each concentration and for all four allergens.

Sensitivity was estimated for each of the reference methods by the number of patients with a positive SPT result (diameter of wheal ≥ 3 mm) and a positive assessment by the reference method divided by the number of all patients with a positive assessment by the reference method.

Specificity was estimated for each reference method by the number of patients with a negative SPT result (diameter of wheal < 3 mm) and a negative assessment by the reference method divided by the number of all patients with a negative assessment by the reference method.

## Results

In total, 435 outpatients were enrolled; 431 patients (mean age, smoker/non-smoker) remained in the Safety Set and 387 were allocated to the Full Analysis Set. Four patients withdrew informed consent and were lost to follow up; these 4 patients were excluded from the Safety Set. Further, 44 patients were excluded from the Safety Set because of invalid tests: 33 patients had a positive saline control reaction and 11 patients had a negative histamine reaction. For demographic data of the Full Analysis Set see Table 1.

**Table 1:** Demographics

	Patients (n = 387)
Age, years (mean, SD)	35.89 (± 10.45)
Gender (n (%))	
Female	234 (60.5)
Male	153 (39.5)
Smokers (n (%))	
Non	221 (57.1)
Ex	79 (20.4)
Current	87 (22.5)
Pets (n (%))	
Yes	156 (40.3)
Formerly, but not at present	36 (9.3)
No	195 (50.4)
sIgE by ImmunoCAP, positive to (n (%))	
Grass pollen	262 (67.7)
House dust mite	187 (48.3)
Birch pollen	252 (65.1)
Mugwort pollen	131 (33.9)
Positive clinical history of allergy to (n (%))	
Grass pollen	237 (61.2)
House dust mite	132 (34.1)
Birch pollen	213 (55.0)
Mugwort pollen	114 (29.5)
Clinical symptoms of allergic (n (%))	
Rhinitis	365 (94.3)
Conjunctivitis	341 (88.1)
Cough/sibilant bronchi	94 (24.3)
Asthma	58 (15.0)
Dermatitis	20 (5.2)
Positive skin prick test result, < 12 months ago to (n (%))	
Grass pollen	290 (74.9)
House dust mite	223 (57.6)
Birch pollen	275 (71.1)
Mugwort pollen	192 (49.6)
Positive family history of relevant allergies, atopic eczema or food intolerance in (n (%)	
Father	61 (15.8)
Mother	93 (24.0)

**Fig. 1:**
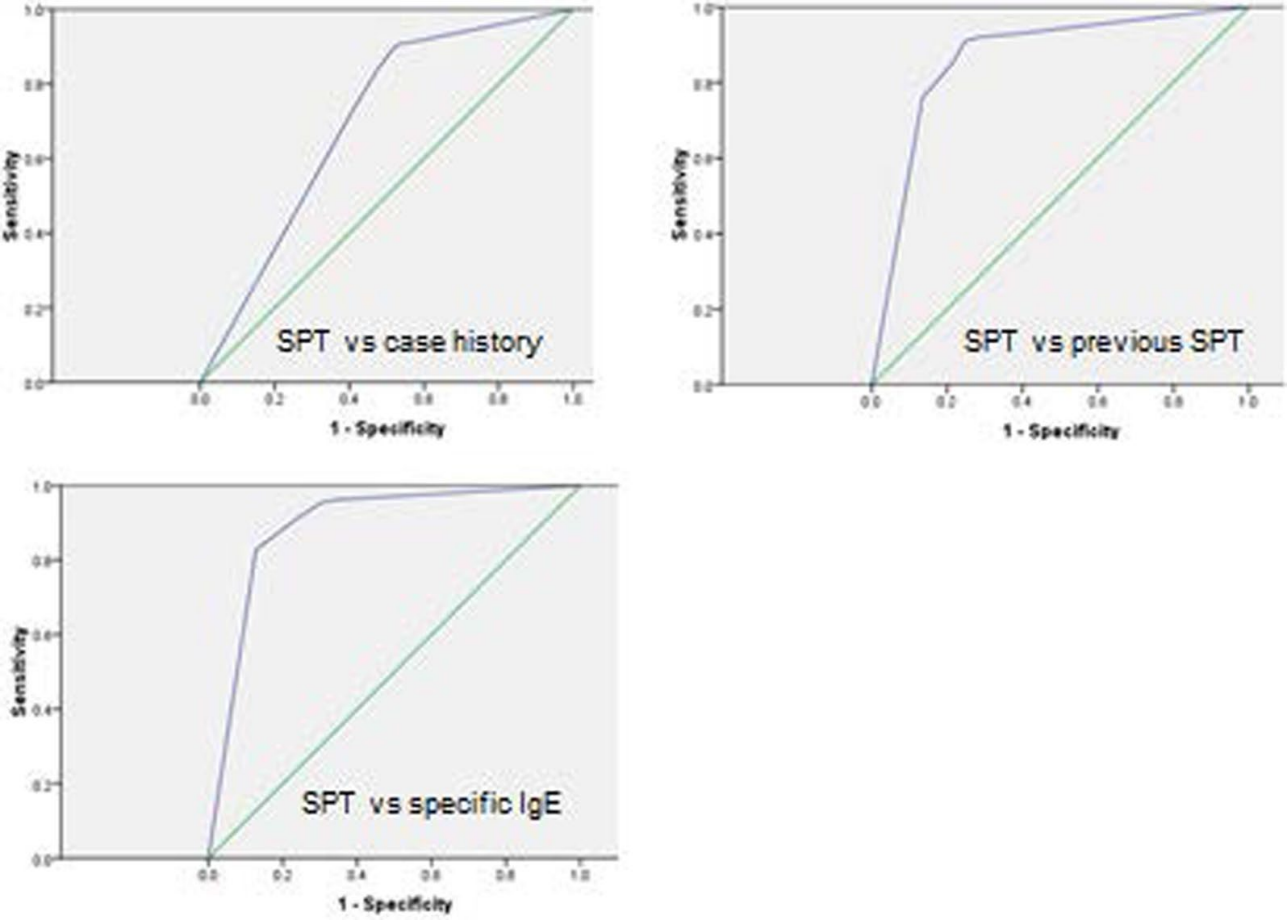
ROC curve for grass SPT versus reference method

The ROC curves illustrate the accuracy of the diagnostic test (Fig. 1). The area under the blue curve shows the accuracy of the test being the combination of correct positive (sensitivity) as well as correct negative (specificity). The area below the ROC curve gives the percentage of times that the test delivers correct results. The green line indicates the area corresponding to 0.5. The combined accuracy would equal 50% and may be considered as an unacceptable test without any discrimination. Thus, the test of the hypothesis H_0_: AUC = 0.5 with a statistically non-significant result would indicate that the diagnostic test gives no information. The larger the area under the ROC curve the more accurate the diagnostic test will be.

In this study, the area under the ROC curve is significantly larger than 0.5 for all allergens and absolute standards in spite of the Bonferroni correction made for multiple testing with α = 0.004167.

The ROC curve analyses showed that all three methods could be used as reference methods for the used SPT solutions (Table 2). The analyses were based on testing the area under the ROC curve and showed that in all cases the area was larger than 0.5. The circulating specific IgE reference method resulted in the largest AUC for all allergens tested (0.80–0.90) followed by the previous SPT (0.70–0.87) and the case history (0.65–0.74) (Table 3).

**Table 2:** ROC curve analyses (AUC = areas under ROC curve)

Allergen	Reference standard	AUC-Area under ROC	p value	95% confidence interval	
Grass pollen	Case history	0.70	< 0.001	0.64	0.75
	Previous SPT	0.87	< 0.001	0.82	0.91
	Specific IgE	0.89	< 0.001	0.85	0.93
House dust mite	Case history	0.74	< 0.001	0.69	0.78
	Previous SPT	0.84	< 0.001	0.80	0.88
	Specific IgE	0.88	< 0.001	0.85	0.92
Birch pollen	Case history	0.69	< 0.001	0.64	0.75
	Previous SPT	0.84	< 0.001	0.79	0.89
	Specific IgE	0.90	< 0.001	0.86	0.94
Mugwort pollen	Case history	0.65	< 0.001	0.59	0.71
	Previous SPT	0.70	< 0.001	0.65	0.76
	Specific IgE	0.80	< 0.001	0.75	0.85

**Table 3:** Sensitivity and specificity for all allergens estimated up against the three reference standards in five different concentrations

Allergen	Reference standard		Concentration of skin prick test solution (SU/mL)				
			5555 (%)	15,666 (%)	50,000 (%)	150,000 (%)	450,000 (%)
Grass pollen	Case history	Sensitivity	72.6	79.3	84.8	87.3	89.0
		Specificity	59.3	53.3	54.7	54.7	50.7
	Previous SPT	Sensitivity	75.9	82.1	87.6	89.3	90.7
		Specificity	87.5	80.2	85.4	84.4	78.1
	Specific IgE	Sensitivity	82.8	89.3	93.5	95.0	95.0
		Specificity	87.2	80.8	80.8	79.2	71.2
House dust mite	Case history	Sensitivity	62.1	73.5	75.0	81.8	90.9
		Specificity	72.9	66.7	62.4	59.6	52.9
	Previous SPT	Sensitivity	61.0	70.9	74.4	79.4	85.2
		Specificity	91.1	86.1	83.5	80.4	70.3
	Specific IjE	Sensitivity	71.7	82.9	85.0	88.8	92.5
		Specificity	91.5	86.5	82.0	77.5	66.5
Birch pollen	Case (Hilary	Sensitivity	71.4	79.8	83.6	85.9	85.9
		Specificity	60.3	52.9	51.7	45.4	48.3
	Previous SPT	Sensitivity	73.8	83.3	85.8	88.7	89.5
		Specificity	85.7	80.0	77.1	69.5	76.2
	Specific IgE	Sensitivity	80.6	90.9	94.8	95.2	96.4
		Specificity	86.7	83.0	83.0	71.9	77.8
Mugwort pollen	Case history	Sensitivity	28.9	43.0	50.0	57.9	66.7
		Specificity	85.7	80.2	75.5	72.5	60.1
	Previous SPT	Sensitivity	29.7	42.2	49.0	53.1	63.0
		Specificity	93.2	89.5	84.7	81.1	68.4
	Specific IgE	Sensitivity	38.2	55.0	60.3	67.2	85.5
		Specificity	91.4	87.9	82.4	79.3	71.5

As anticipated it was found that the sensitivity of the SPT increased with increasing concentration of the SPT solution and that the specificity decreased for all four allergens and three reference methods (Table 3). For the reference method ‘circulating specific IgE’, the sensitivity as well as specificity achieved values over 80% for grass pollen and birch pollen at concentrations of 5555 SU/mL, 16,666 SU/mL and 50,000 SU/mL and for house dust mite at concentrations of 16,666 SU/mL and 50,000 SU/mL. For mugwort pollen, the highest sensitivity (60.3%) with a specificity of at least 80% was observed for the concentration 50,000 SU/mL.

For the reference method ‘previous SPT’, the following concentrations were > 80% regarding sensitivity as well as specificity: 6-grass pollen 16,666 SU/mL, 50,000 SU/mL and 150,000 SU/mL and birch pollen 16,666 SU/mL. For mite and mugwort, sensitivity and specificity above 80% were not found.

In general, the ‘case history’ as reference method showed the lowest values for sensitivity and specificity for all allergens when compared to the other two reference methods. Based on these analyses, the reference method of choice is ‘circulating specific IgE’ for all four allergens. This reference method has the highest sensitivity with a specificity of at least 80%. At the 50,000 SU/mL concentration, the sensitivity for grass pollen, birch pollen, house dust mite and mugwort was 93.5%, 94.8%, 85.0% and 60.3%, respectively.

No serious adverse events were reported. The reported adverse events were all expected and only one non-serious systemic adverse event was reported (dizziness occurred 30 min after the waiting period). The event lasted for 1 min and no therapeutic measures were necessary.

## Discussion

The objective of this study was to find an optimal concentration with respect to specificity and sensitivity for SPT solutions of the four allergens 6-grass pollen mix, house dust mite *Dermatophagoides pteronyssinus*, birch pollen and mugwort pollen in a large multicenter study across different geographical areas in Germany. These allergens are part of the Pan-European skin prick test panel based on the GA^2^LEN study which are recommended to be used throughout Europe [12]. This was investigated according to the latest recommendations of the Committee for Proprietary Medicinal Products (CPMP) in the ‘Points to consider on the Evaluation of Diagnostic Agents’ and the ‘Guideline on Clinical Evaluation of Diagnostic Agents’ [5, 6]. Because an overall accepted ‘absolute standard’ is lacking for the diagnostic test system in the field of allergology [﻿13﻿], the evaluation was done by the method of the ROC with three different reference methods. This method was first described by Wheeler and co-workers in 1996 [14] for timothy grass pollen using ‘case history’, ‘challenge tests’ and ‘RAST test’ as reference methods. The number of patients tested was markedly smaller (n = 53) compared to this study including 435 patients in the safety set. A working group with A. W. Wheeler also described ROC analysis for identifying the most appropriate concentration range for house dust mite (*D. farinae*, *D. pteronyssinus*), cat and dog epithelia SPT solutions [15].

The reference methods used in our study were ‘circulating specific IgE’, the results of a ‘previous SPT’, and the ‘case history’.

The ROC curves showed that all three reference methods could be used for SPT solutions to find the optimal concentration for diagnostics. The circulating specific IgE as reference method resulted in the largest AUC for all allergens tested; for birch pollen the AUC was as high as 0.9. We therefore defined ‘circulating specific IgE’ as our preferred reference standard for determination of sensitivity and specificity of the SPT solutions. Good concordance has been identified between a positive SPT result and serological specific IgE for most of the aeroallergens and the ImmunoCAP™ is the assay that has been studied most extensively [7, 8, ﻿13]. SPT and specific IgE immunoassays provide confirmation of sensitization by detection of specific IgE antibodies, but not necessarily the presence of allergic symptoms. Sensitivity and specificity for house dust mite were inferior to results of birch pollen and grass pollen (six-grass pollen mixture), which might depend on the allergen content in skin prick solution. We know by now, that compared to grasses and birch many more allergens in house dust mite generate sensitizations. r Der p 23 might be a clinically relevant allergen in house dust mite allergy for some individuals [16]. If a high number of allergens is responsible for the sensitization against one species, there might be higher variations in the compositions and the amount of these allergens for SPT and serologic immunoassay.

Sensitivity for mugwort pollen displayed the weakest sensitivity for SPT and specific IgE compared to the other aeroallergens, which is phenomenon observed by Lee likewise [﻿17﻿]. It might depend on the raw material used for skin prick test solution, as shown in immunoblotting studies [﻿17﻿].

Differences in the ‘previous SPT’ and the SPT performed in this study could be caused by the different prick test solutions of various manufacturers used, as commercially available allergen solutions are not comparable and show high variety in allergen composition and content of allergens [18–21]. Furthermore, the reproducibility of skin prick test is influenced by the technique used [22] or the prick test device [23] and interpretation of skin reaction influence results  [1, 24]. The ‘previous SPT’ was performed under the usual conditions of a medical practice while the SPT in this study was performed under highly standardised conditions and was additionally applied blinded.

Several patients showed a false positive reaction to saline control solution. False-positive skin prick results may be due to symptomatic dermographism or might be induced by “irritant” reactions or a nonspecific enhancement from a nearby strong reaction [﻿1﻿]. Notably the latter cannot be reassessed due to the blinded study design.

In former studies, diameters of wheals were measured. More recent studies rely on the exact wheal area, as in our study, recorded by outlining the circumference [25]. Furthermore, it has to be taken into account, that sensitizations to aeroallergens, measured by skin prick test or specific IgE, may precede symptomatic allergy. Prospective studies show that 30–60% of such subjects become allergic depending on the type of allergen tested and the time to follow-up [3].

Specific IgE antibodies may be present without clinical symptoms of allergy and some patients with clinically manifested allergy have negative test results when using objective measures [24, 26]. Patients history, when requested retrospectively is subject to individual remembrance and less reliable than prospective, seasonal concomitant documentation. This might explain why the reference method ‘case history’ showed the lowest comparability to SPT in this study. Our result is in accordance with data of Smith and co-workers [27] showing that the accuracy of an assessment of the patient’s allergic status can be improved by adding a SPT to a structured allergy history alone. Many studies show that both testing methods, specific IgE and SPT, should complement  each other for if only one method is used, about 25% of sensitized patients would be missed [28–30].

The sensitivity of specific IgE immunoassays comparing SPTs has been reported to range between less than 50% to greater than 90%, with an average of 70% to 75% depending on the allergen tested [24, 30–34]. Our data, measured with the ImmunoCAP™ system, are in accordance with these reports: the highest sensitivity including a specificity of at least 80% was observed for the SPT solution of 50,000 SU/mL for all four allergens tested.

Referring to ROC analysis, requiring an AUC of at least > 0.5, all allergen concentrations examined, could be used, accepting a reduced specificity using the highest concentrations. Similar findings were described by Focke et al., showing that even a great variation in content of allergens in test solutions gives a positive SPT result in allergic patients [﻿20﻿].

The investigational product was very well tolerated and there were no safety concerns.

## Conclusion

We used clinical case history, previous SPT and specific IgE measurement as reference methods for the assessment of sensitivity and specificity of one manufacturer’s SPT solutions (6-grass pollen mixture, house dust mite (*D. pteronyssinus*), birch pollen and mugwort pollen). The comparison with specific IgE resulted in the largest AUC. The highest sensitivity based on a specificity of at least 80% was observed for the SPT solution of 50,000 SU/mL. This is the standard concentration of the manufacturer’s SPT solutions registered in several European countries.

The decision for an optimal causal treatment as allergen-specific immunotherapy should be based on objective measurements as SPT or specific IgE in combination with a medical investigation and case history.

## Abbreviations

SPT: skin prick test
SU: standardised units
AUC: area under curve
ROC: receiver operating characteristics
Ig: immunoglobulin
